# Senescence induction; a possible cancer therapy

**DOI:** 10.1186/1476-4598-8-3

**Published:** 2009-01-08

**Authors:** Matilde E LLeonart, Ana Artero-Castro, Hiroshi Kondoh

**Affiliations:** 1 Pathology Department, Fundació Institut de Recerca Hospital Vall d'Hebron, Passeig Vall d'Hebron 119-129, 08035 Barcelona, Spain; 2 Department of Geriatric Medicine, Graduate School of Medicine, Kyoto University, 54 Kawahara-cho, Shogoin, Sakyo-ku, Kyoto, 606-8507, Japan

## Abstract

Cellular immortalization is a crucial step during the development of human cancer. Primary mammalian cells reach replicative exhaustion after several passages *in vitro*, a process called replicative senescence. During such a state of permanent growth arrest, senescent cells are refractory to physiological proliferation stimuli: they have altered cell morphology and gene expression patterns, although they remain viable with preserved metabolic activity. Interestingly, senescent cells have also been detected *in vivo *in human tumors, particularly in benign lesions. Senescence is a mechanism that limits cellular lifespan and constitutes a barrier against cellular immortalization. During immortalization, cells acquire genetic alterations that override senescence. Tumor suppressor genes and oncogenes are closely involved in senescence, as their knockdown and ectopic expression confer immortality and senescence induction, respectively. By using high throughput genetic screening to search for genes involved in senescence, several candidate oncogenes and putative tumor suppressor genes have been recently isolated, including subtypes of micro-RNAs. These findings offer new perspectives in the modulation of senescence and open new approaches for cancer therapy.

## Background

**Figure 1:**
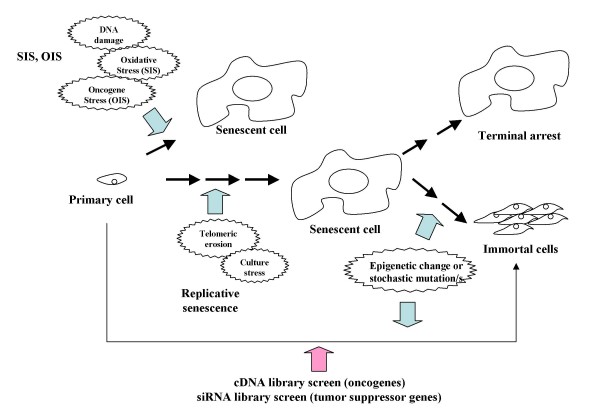
**Primary cells reach replicative senescence after several passages in culture**. The passage number where they reach senescence is affected by the tissue conditions, etc. In addition, senescence can be forced to arise upon SIS. A senescence cell can remain arrested for a long period of time. Alternatively, epigenetic changes and/or stochastic mutation/s can lead it to escape from senescence.

Primary mammalian cells reach proliferative exhaustion after serial passage in culture, resulting in a permanent and irreversible cell-cycle arrest [1]. This process, termed replicative senescence, is characterized by a drastic phenotypic change in the cells, compared to their proliferating counterparts [2]. The precise number of replicative doublings exhibited by cultured cells before they reach senescence depends on the species from which the cells are derived, the tissue of origin, and the age of the donor organism. Senescent cells are characterized by expression of β-galactosidase, PAI-1 overexpression (plasminogen activator protein 1) and altered cell morphology characterized by giant cell size, increased cytoplasmic granularity and a single large nucleus (Figure 1). Senescent cells, arrested in G1 phase of the cell cycle, remain viable and metabolically active and possess a characteristic transcriptional prolife that distinguishes them from quiescent cells [3]. In order to form tumors, incipient cancer cells must breach this senescence barrier that normally limits their proliferative potential.

Approximately one-fourth of people in the developed world experience cancer during their life time. Cancer cells display several hallmarks that can be distinguished from those of normal counterparts. These include immortalization or bypass of senescence, evasion of apoptosis and anti-growth signals, growth factor independence, enhanced glycolysis, anchorage-independence, resistance to contact inhibition, angiogenesis, degradation of matrix components, invasion, migration, etc. These properties may provide good targets for anti-cancer drugs, such as DNA damaging, anti-angiogenic, differentiation-inducing, or apoptosis-inducing agents. It is likely that senescent cells will ultimately be cleared by phagocytosis, although it has not beeen clearly demonstrated for the senescent state [4]. For example, senescent neutrophils might face phagocytosis through an unknown mechanism [5] and focal enrichment of lysosome-related β-galactosidase activity at autodigestive vacuoles showed that aged human fibroblast arrested in replicative senescence might eliminate themselves by autophagy [6].

Interestingly, senescent cells have recently been detected during tumorigenesis in mouse models and in human tumors, particularly in benign lesions [7,8], and their appearance is possibly relevant to tumor progression. There are examples of *in vivo *senescent cells that may reside for years in tissue, such as the senescent melanocytes of moles of nervi. Other senescent cells can be rapidly removed as tumors progress, for example in the case of liver carcinomas [9,10].

The cellular senescence observed in tissue culture might constitute a good model for understanding the significance of senescence *in vivo*. Recent attempts at screening cells for the ability to bypass senescence have identified several putative novel oncogenes and tumor suppressor genes. Genes whose ectopic expression results in replicative senescence are considered potential oncogenes [11] and are overexpressed in some types of cancer [12,13]. On the other hand, genes whose inactivation results in cellular immortalization are potential tumor suppressor genes [14,15] and are found to be downregulated in tumor-resident tissues [16,17]. Therefore, senescence is considered an anti-tumorigenic mechanism for avoiding indefinite cell proliferation when a genetic alteration has occurred. An important issue to consider in a senescence-based therapy is the fact that the ultimate destiny of a senescence cell is its terminal arrest (Figure 1). In this regard a metabolically arrested cell is not responsive to mitogenic signals and it is quite unlikely that it resumes proliferation. If senescent cells are able to reside for years in tissue, an epigenetic change could lead to escape from senescence. Therefore future research should also focus on discovering senescence markers that can be used for monitoring *in vivo *the presence/absence of senescent cells. Recent progress in the biology of cellular senescence provides another clue to understanding the mechanism of cancer progression, as well as to development of new anti-cancer drugs. This fact suggests that senescence-inducing mechanisms might be applicable as cancer therapies in the future.

### Oncogenes and tumor suppressor genes

The best characterized example of oncogene-induced senescence is the response of normal fibroblasts to expression of an activated allele of H-ras (H-ras^V12^). Normal ras proteins are important for transducing mitogenic signals in the cell and are mutated to constitutively active forms in approximately 20% of human cancers [18]. These activated alleles contribute to transformation in human cancer by increasing proliferation, and invasion of tumors, as well as desensitizing cells to apoptosis [19]. All human normal cells with intact p53 and retinoblastoma (Rb) pathways enter into senesce in response to RAS. Expression of viral oncoproteins that disrupt these pathways, such as simian virus 40 (SV40) T Ag, human papillomavirus E6 or E7, or adenovirus E1A, block senescence and cooperate with RAS to transform cells [20]. In contrast than in human cells where the p16/Rb pathway plays a more significant role, in murine cells, an intact p53/Arf pathway is required for RAS-induced senescence.

**Figure 2:**
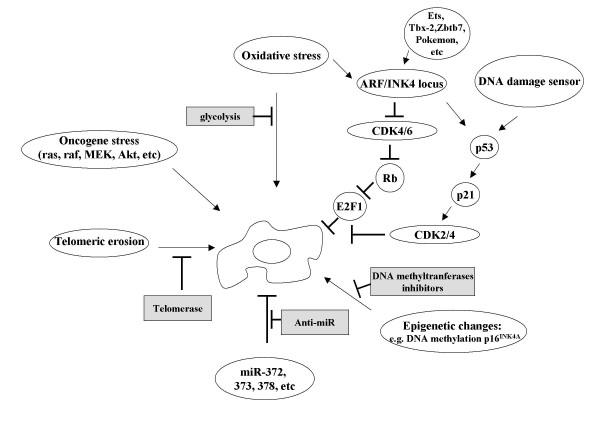
**Senescence-inducing factors as targets of cancer therapy**. Details are described in the text.

It is of note that SIS and OIS can be bypassed partially by inactivation of tumor suppressors, including the p53 or Rb axis, implicating the involvement of several tumor suppressor genes in these stress-induced senescence processes (Figure 2). In cancerous cells or tissues, tumor suppressor genes may be inactivated by either deletion of one or both alleles, promoter methylation, splice-site mutations or nonsense mutations, or a combination of these. Alternatively, mutations in tumor suppressor genes can provide a dominant negative protein that interferes with the wild-type protein produced by the other allele, as is the case for several p53 mutants. Such genetic alterations result in a complete absence or partial reduction of the tumor suppressor protein, conferring a selective advantage in clonal selection for tumor progression.

The presence of senescent cells has been demonstrated *in vivo *to positively correlate with aging, illustrating that the *in vitro *observation of replicative senescence is applicable to the events *in vivo *[21]. Recently, a significant *in vivo *role for the process of senescence was demonstrated. Several groups have shown that benign tumors contain senescent cells and that these cells fully disappeared in the malignant counterparts of the described tumors. As the process of senescence is observed both *in vitro *and *in vivo *[2,7,22], tumor suppressor activation can be another target of cancer therapy *in vivo*. Promising results have been recently reported in a murine model where complete tumor regression could be provoked by p53 activation [10,23,24]. *In vitro *restoration of p53 function triggers dramatic and rapid induction of p53 target genes, as well as apoptosis. While several groups have reported similar effects, it is not just p53 status itself that determines therapeutic efficacy of p53 restoration, but also the status of p53-activating signals that pre-exist, or can be induced, in tumor cells. Although this situation would not be exactly the same as that which occurs in human cancer (where p53 is affected or the p53 pathway is corrupted), regression of these tumors was closely correlated with the presence of senescent cells. Thus, these studies provide the first evidence that senescence induction *in vivo *can be a crucial mechanism of tumor clearance. It would be worthwhile to further investigate whether p53 activating chemicals, such as quiacrine, might be beneficial for cancer patients.

The ARF/INK4a (Alternative Reading Frame) locus encodes two distinct tumor suppressors, ARF (p14^ARF ^in humans and p19^ARF ^in mice) and p16^INK4A^. p16^INK4A ^and ARF respectively regulate the pRB and p53 pathways of senescence and tumor suppression [25]. p16^INK4A ^binds and inhibits cyclin-D-dependent kinases CDK4 and CDK6. Such kinases have oncogenic potential because they phosphorylate the retinoblastoma family of tumor suppressors Rb, p107 and p130, which are negative regulators of the cell cycle (Figure 2). ARF is an antagonist for Mdm2, which regulates p53 stability through its p53-degrading ubiquitin ligase activity. ARF sequesters hmdm2 through its translocation to the nucleolus and the final consequence is p53 activation and stabilization.

During replicative senescence *in vitro*, the accumulation of these tumor suppressors (p53, ARF, INK4A) or their downstream targets (p21^Cip1^) is observed [26]. Oncogenic insults also upregulate these genes, including p53, ARF, INK4, and p21^Cip1^. It is noteworthy that the ectopic expression of these tumor suppressors (ARF, INK4, p21^Cip1^) can provoke a senescence-like phenotype in primary or immortalized cells.

All of these senescence-inducing tumor suppressor genes may also be targets of anti-cancer drugs. Several ARF transcriptional repressors have been identified in a senescence bypass screen, including TBX2 [27] and Zbtb7 or pokemon [28]. INK4 is known to be regulated by the Ets transcriptional family. These regulators for ARF/INK4 might be good candidates for anti-cancer drugs through modulation of their senescence effect. However, as is often the case with p53, the inactivation of the ARF/INK4a locus by complete deletion or by aberrant promoter methylation is very common, being present in about 30% of all known types of malignancies. Presumably, in cancer cells harboring genetic inactivation of this locus, the modulation of ARF/INK4 function would be less effective as cancer treatment.

Alternatively, a CDK inhibitor may be a good tool, as both the ARF/p53 and INK4/Rb axis exert their tumor suppressing function partially via inactivation of CDK4. Several small chemical modulators for CDK kinases are currently under investigation in clinical trials. So far, these are not CDK4-specific inhibitors, but rather pan-CDK inhibitors, such as Fravopiridol, UCN-01, Roscovitine, and BMS387032. The anti-cancer effects of these compounds mainly result in induction of apoptosis rather than provocation of senescence. This is possible because CDKs play additional and essential roles beyond cell cycle control, including transcription, DNA repair, migration, and secretion.

### Telomeres

Human telomeres consist of tandem repetitive arrays of the hexameric sequence TTAGGG. Overall telomere sizes range from 15 kb at birth to <5 kb in old individuals, as telomeres become progressively shorter with every round of cell division in normal cells [29]. The length of telomeric DNA decreases progressively in primary cells as they replicate. The ends of telomeres are protected and regulated by telomere-binding proteins and form a special lariat-like structure called the T-loop. Telomerase is a complex cellular ribonucleoprotein enzyme composed of a number of distinct subunits responsible for adding telomeric repeats to the 3'ends of chromosomes. It has two major components, an enzymatic human telomerase transcriptase catalytic subunit hTERT, and a RNA component hTERC. Telomerase uses its integral RNA component as a template in order to synthesize telomeric DNA directly onto the ends of chromosomes. Primary normal human cells stably expressing transfected telomerase can divide indefinitely, providing direct evidence that telomere shortening has a causal effect on replicative senescence [30]. The enzyme is normally expressed in very few primary cells such as embryonic stem cells [18]. Interestingly, some viral oncoproteins are able to modulate telomerase expression and inhibition of telomerase limits the growth of human cancer cells [19,20]. Telomerase is also present in adult male germline cells, but it is undetectable in most normal somatic cells, with the exception of proliferative cells of renewal tissues. It has been reported that several human diseases, such as dyskeratosis, congenital or aplastic anemia, are caused by the mutations in genes encoding components of telomerase or telomere-binding proteins. These mutations cause low telomerase activity, accelerated telomere shortening, and diminished proliferative capacity of hematopoietic progenitors.

Cellular senescence is a process that is triggered not only by telomeric erosion but also by other forms of stress. Several factors provoke senescence in a telomere-independent manner. Stress-induced senescence (SIS) can be induced by DNA damage, ionizing radiation such as X-rays or UV, and oxidative stress. Oncogene-induced senescence (OIS) can be induced by oncogenic activation. The most well-defined model of oncogene induced-senescence is shown by an activated allele of the *ras *gene (ras^V12^) whose overexpression is accompanied by a concomitant accumulation of p53 and p16^INK4A ^proteins [21]. Interestingly, it has been shown that adenomas from mice expressing a single activated K-ras allele undergo senescence *in vivo *[7]. Several oncogenes are known to induce a senescence response upon overexpression: ras, raf, MEK, Akt, E2F1/3, mos, PTEN, NF1, Stat5, KLF-4, and Runx.

When senescence is induced by a stimuli such as oncogenic ras or raf, by epigenetic changes, or by oxidative damage, the ectopic expression of hTERT cannot confer the senescence-bypassing effect [31]. However, in several cases of telomere-independent senescence, the initiating event can be triggered by a common mechanism. For example, cellular damage caused by these stresses would be recognized by cellular sensors of the DNA damage checkpoint apparatus, leading to the activation of cell cycle checkpoint responses, including ataxia telangiectasia mutated ATM-Chk2 kinase and ATR/Chk1 signaling, and recruitment of DNA repair foci. Tumor cells in clinical specimens from various tissues such as breast and lung carcinomas often show constitutive activation of DNA damage signaling, including activated forms of checkpoint kinases ATM and Chk2, phosphorylated histone H2AX and p53, and foci formation by proteins such as 53BP1. In this way, phosphorylated p53 is stabilized and protected from destruction. Indeed a single dsDNA break occurring anywhere in the genome seems sufficient to induce a measurable increase in p53 levels. The cellular effects of p53 are mediated by its ability to transactivate specific genes, including p21^Cip1^, 14-3-3-σ or Puma [32], or its ability to induce downregulation of specific proteins such as CDK4 and cyclin E [25]. Activation of DNA damage signaling may be followed by changes in chromatin structure as the formation of senescence-associated-heterochromatin foci (SAHFs) [26]. SAHFs accumulate during oncogene-induced senescence and are thought to stably suppress the expression of E2F target genes by recruiting Rb and heterochromatin proteins.

Telomerase activity is detected in approximately 90% of all malignant tumors in comparison with their normal counterparts. This suggests that telomerase may be a suitable target for anti-cancer drugs. It was demonstrated that siRNA against telomerase or overexpression of a dominant-negative mutant of telomerase abolished telomerase activity and resulted in entry of cells into crisis [33]. Several clinical trials targeting telomerase are ongoing for advanced cancer patients. These include immunotherapy (a vaccine against telomerase), inhibitory compounds against telomerase activity (a telomerase template antagonist), and the modulation of telomeric structure (telomestatin). The expected outcome of telomerase inhibition should not only be to slow tumor growth, but also to diminish the possibility of induced apoptosis, for the following reasons: The tumor suppressor p53 is presumed to sense dysfunctional telomeres as damaged DNA, whereupon it elicits the senescence response at least in part by increasing expression of the cell cycle inhibitory protein, p21^Cip1^. This, in turn, prevents the inactivation of p53 [34,35]. Thus, in p53-intact cells, accelerated telomeric shortening by telomerase inhibitors could be sensed by the p53 pathway and would be followed by initiation of massive apoptotic death.

Telomerase activity is a useful prognosis indicator of the outcome of neuroblastomas that are usually encountered in very young children. Telomerase activity should be incorporated into the clinical investigation of each individual neuroblastoma at the time of diagnosis because its mere presence/absence is sufficient basis for predicting disease outcome [36]. Therefore, neuroblastomas would be good candidates for the above-mentioned telomerase inhibitors.

### Micro-RNAs

Micro-RNAs (miRNA) are a novel class of non-protein encoding genes that have appeared to play an important role in the post-transcriptional regulation of gene expression [37]. Mature miRNAs are double-stranded RNA molecules ~22 nucleotides in length that are the product of several processing steps of PolII-transcribed primary RNA transcripts. The final step in the miRNA pathway is the loading of one of the mature RNA strands into the RNA-induced silencing complex (RISC) that subsequently binds to a mRNA with a certain degree of complementarity, often in the 3'UTR of the mRNA. Depending on the degree of complementarity, miRNAs are involved in sequence-specific degradation of mRNAs or inhibition of protein translation [38]. One miRNA can control the expression of many different genes, and it is speculated that 20–30% of total gene expression may be regulated by miRNAs. Bioinformatics approaches have identified 300 human miRNA genes, but more recent work has predicted the number to be closer to 1,000 [39]. Recent studies have shown that miRNA expression profiles are different between normal and tumor tissues [38,39]. Interestingly, downregulation of subsets of miRNAs is a common finding in some tumors. The discovery that miRNA silencing could revert the tumorigenic phenotype of the colon cancer cell line HCT116 unveils a novel regulatory mechanism in cancer proliferation [40].

Recently, several laboratories have reported that members of the miR-34 family are direct targets of p53, which induces apoptosis, cell cycle arrest and senescence [41-43]. Several miRNAs are significantly induced by p53, including miR-16, which targets the oncogene bcl-2; and let-7, which downregulates ras and HMGA2. Among the mi-RNAs repressed by p53 is miR-221 which downregulates p27.

In a genetic screen to identify miRNAs characterized by their ability to bypass senescence induced by oncogenic Ras (OIS), miR-372 and miR-373 were identified [44]. This study was performed in partially immortalized IMR90 fibroblast. These miRNAs are considered novel oncogenes participating in the development of human testicular germ cell tumors by numbing the p53 pathway and promoting tumorigenic growth in the presence of wild-type p53. Importantly, the fact that miR-373 is able to form foci in soft-agar assays shows its transforming capability. On the contrary, introduction of miR-34a and miR-34b/c into primary human diploid fibroblasts induced cellular senescence [45]. Tumor cells also showed signs of senescence after introduction of ectopic miR-34a [46]. Downregulation of miR-138 is associated with overexpression of telomerase and the acquisition of malignant behavior in human anaplastic thyroid carcinoma cell lines [47]. Thereby, it is expected that targeting miR-138 would be useful as a diagnostic tool or might contribute to the development of new strategic treatments for specific kinds of carcinomas as already suggested for miR-378 [48].

### Oxidative stress

Reactive Oxygen Species (ROS) can produce serious damage in cells [49]. Cumulative oxidative damage causes or accelerates senescence, while immortalized cells are resistant to the senescence effect of oxidative damage. The most dramatic observations have been reported when cells have been exposed to different oxygen concentrations. When oxygen is reduced from 20% to 1–5%, human diploid fibroblasts increase their replicative life-span between 20–50% [49]. In this sense, the modulation of oxidative stress could be another target of cancer therapy.

It has been reported that enhanced glycolysis via ectopic expression of a glycolytic enzyme (phosphoglycerate mutase; PGM) immortalizes primary MEFs, protecting them from oxidative damage [50]. Enhanced glycolysis is a well-known property of most cancerous cells and tissues, commonly referred to as the Warburg effect. This property is well utilized in clinical practice for the detection of metastatic tumor mass through positron-emission scanning of 2-[^18^F]fluoro-2-deoxy-D-glucose [51]. Thus, inhibition of glycolysis may represent an alternative therapy for cancer treatment. PGM inhibition in primary MEFs induced premature senescence. Indeed, the PGM inhibitor MJE3 has been identified as the most potent anti-proliferative reagent via chemical screening in breast cancer cells [50].

Currently several glycolitic inhibitors with promising results in cultured cells and animal models are being tested in clinical trials. For example, preclinical studies include 3-Bromopyruvate (3-BrPA) in clinical trials (I/II). A compound at a further stage of testing is 2-Deoxyglucose (2-DG), in clinical trials (II/III). 3-BrPA inhibits hexokinase causing a depletion in ATP leading to massive cell death in those cells with respiration defects. 2-DG is an agent that is phosphorylated by hexokinase, but it cannot be further metabolized and thus blocks glucose metabolism [52]. 2-DG has shown promising results enhancing significantly the anti-cancer activity of adriamycin and paclitaxel in mice bearing human osteosarcoma and non-small-cell lung cancer xenographs [53].

Collectively, senescence induction would be a good tool for cancer treatment in the future. A personalized genetic test of the status of several oncogenes and tumor suppressors, including ras, p53, p16^INK4A^, ARF and hTERT, should be carefully examined before senescence-induction treatment. Once the status of these senescence-associated genes is known, it should be easier to predict the sensitivity or resistance of a tumor to senescence-induction treatment.

### Epigenetics

Epigenetic changes occur constantly in wild-type mammalian cells as a dinamic feature that involves the activation/inactivation of numerous genes. Examples of epigenetic changes are: methylation of DNA, histone deacetylation, ubiquitination and phosphorylation. Overall these changes, DNA methylation is considered to play an essential role in cellular senescence and aging [54]. It has been demonstrated that the methylation level in the genome decrease gradually during SIS as well as replicative senescence. This has been associated with the reduction in the expression of the methylation enzyme DNMT1. Such changes reflect global hypomethylation as a distinct feature of senescent cells [55]. SIS share some features of replicative senescence, such as basic biological characteristics and global hypomethylation while there are slight differences in the profile of methylation-associated enzyme expression. Aging is accompanied by changes in DNA methylation in tumor suppressor genes and by stochastic methylation events through the genome. Thereby, aging-related random methylation has been proposed to occur as a result of failure of methyltransferase activity, and/or exposure to environmental factors and health of the individual [56].

The pattern of altered gene expression or epigenetic changes is of major importance in common malignancies. DNA methylation patterns are severely affected in cancer. p16^INK4A^, possibly after p53, is the most important tumor suppressor gene altered in human cancer. Methylation of the p16^INK4A ^promoter implies p16^INK4A ^silencing and subsequently the loss of the p16^INK4A ^protein. p16^INK4A ^promoter methylation occurs in specific cancers as breast and hepatocellular carcinoma. Importantly, promoter hypermethylation of the p16^INK4A ^gene is associated with poor prognosis in recurrent early-stage hepatocellular carcinoma [57]. However, although a great number of tumor suppressor genes are hypermethylated in regions rich in CpG (CpG island); paradoxically the general pattern of the cancer genome is a general DNA hypomethylation. Interestingly, epigenetic changes that occur in cellular senescence *in vitro *in HMECs constitute again a good model to recapitulate the adquisition of premalignant lesions *in vivo *[58]. For example p16^INK4A ^inactivation by promoter methylation is one early event that contribute to HMEC immortalization. Interestingly, our group have recently identified in a loss-of-function genetic screen the methylation enzyme S-adenosylhomocysteine hydrolase (SAHH). SAHH inactivation bypass replicative senescence and induces immortalization in primary murine cells affecting both the p53 and pRb pathways [17]. In addition, SAHH has been found altered in human tumors at mRNA and protein levels suggesting that it could be a putative novel tumor suppressor gene [17]. The fact that SAHH is able to modulate senescence reinforces the importance of methylation enzymes in immortalization and cancer development.

In order to stimulate the expression of those tumor suppressor genes silenced in cancer, much attention has recently been focused on developing small molecule inhibitors of DNA methyltransferases that could be used as anti-cancer drugs such as 5-Azacytidine and 5'-Aza-2'-deoxycytidine (decitabine). Surprinsingly, they have been quite effective in leukemias, however in solid tumors they have not been very successful. Importantly, the lack of p53 inducibility of apoptotic genes, has been restored by treatment with 5-Aza-2'-deoxycytidine suggesting that epigenetic cancer therapy is possible for some cancers in combination with forced p53 activation [59]. Importantly, while the acquisition of spontaneous mutations that disable p53 or pRB in a resting cell without DNA replication seems rather unlikely, epigenetic changes might occur in senescent cells. If this represents an additional barrier to take into account before applying a senescence induced anti-cancer therapy, it deserves deeply and extensive investigations.

## Conclusion

**Figure 3:**
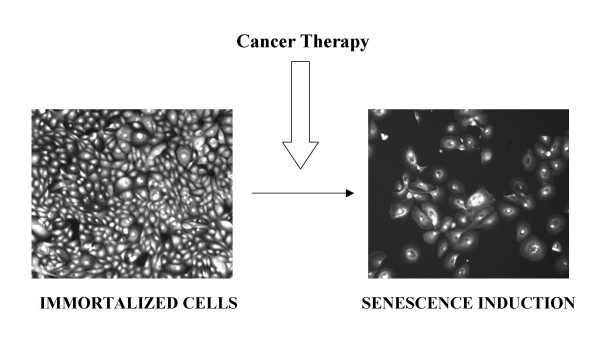
**Phenotypic aspect of senescence induction from human mammalian epithelial cells (HMEC) immortalized with the TERT telomerase (left panel)**. HMEC are induced to enter into senescence (right panel). HMEC are fixed and stained with Cell Mask to visualize cell morphology.

Cellular senescence has become an attractive therapeutic concept because mimics programmed cell death by excluding cells from active progression through the cell cycle. As an intact apoptotic machinery is unavailable in most established malignancies, a senescence-induced mechanism emerges as a back-up program to substitute for or to reinforce an insufficient apoptotic response. Importantly, the therapeutic potential of senescence induction strongly relies on the irreversibility of this process. Although, it has not been formally tested whether drug-inducible senescence is a complete reversible process, the acquisition of spontaneous mutations that disable p53 or pRB in a resting cell without DNA replication seems rather unlikely. Thereby it would be very interesting for preclinical investigations to explore future therapies which directly prompt a clear senescence response as those observed *in vitro *(Figure 3). It would be tempting to speculate that the combination of a senescence induced therapy and a conventional therapy might cooperate to entirely abolish cancer.

## Abbreviations

HMEC: human mammalian epithelial cells; MEF: mouse embryonic fibroblast; SIS: stress induced senescence; OIS: oncogene induced senescence

## Competing interests

The authors declare that they have no competing interests.

## Authors' contributions

MELL was responsible for writing, revising for intellectual content and final approval of the manuscript. AAC designed some sections of the manuscript including figures.

HK was responsible for writing, revising and final approval of the manuscript.
